# Radiation-Associated Herpes Zoster: A Clinical Case

**DOI:** 10.7759/cureus.75857

**Published:** 2024-12-17

**Authors:** Teodor Aleksiev, Veselin Popov, Hristo Dobrev

**Affiliations:** 1 Dermatology and Venereology, Medical University of Plovdiv, Plovdiv, BGR; 2 Clinical Oncology, Medical University of Plovdiv, Plovdiv, BGR

**Keywords:** breast cancer, herpes zoster, radiodermatitis, radiotherapy (rt), : thermography

## Abstract

Herpes zoster (HZ) is a viral infection caused by the reactivation of endogenous and latent varicella-zoster virus that remains dormant in the cranial nerve or dorsal root ganglia. HZ occurs in a portion of the general population, with a higher incidence observed in high-risk individuals. Patients with impaired immunity, including human immunodeficiency virus infection, organ transplantation, old age, and cancer-related treatments such as chemotherapy (CT) and radiotherapy (RT) were found more prone to HZ infection. We present a case of a 50-year-old patient who underwent a surgical excision of an invasive ductal carcinoma of the right breast. Following 15 fractions of RT, the patient presented with HZ appearing in the radiation field. The patient was treated successfully with Acyclovir, and RT was continued while on maintenance therapy with antiviral drugs. This case presents the importance of early diagnosis and the right choice of treatment in cancer patients and HZ due to the higher risk of complications and further development of the primary condition.

## Introduction

Herpes zoster (HZ) is a viral infection caused by the reactivation of endogenous and latent varicella-zoster virus that remains dormant in the cranial nerve or dorsal root ganglia [1]. It is characterized by dermatomal unilateral painful vesicular rash, most commonly preceded by a burning sensation or pain, which may appear on different areas of the body. The most common localization is the chest, head, and neck. It is less commonly seen on the limbs, hands, or feet [2]. The incidence of HZ is between 10-20%; however, in high-risk individuals, it can be observed in up to 50%. Patients with different immunodeficiency conditions, including cancer-related treatments such as chemotherapy (CT) and radiotherapy (RT), were found to be more prone to HZ infection [2]. Different types of cancer are a predisposing factor for HZ. Patients with hematological malignancies are at a higher risk of HZ development compared to patients with solid tumors [3-6].

## Case presentation

**Figure 1:**
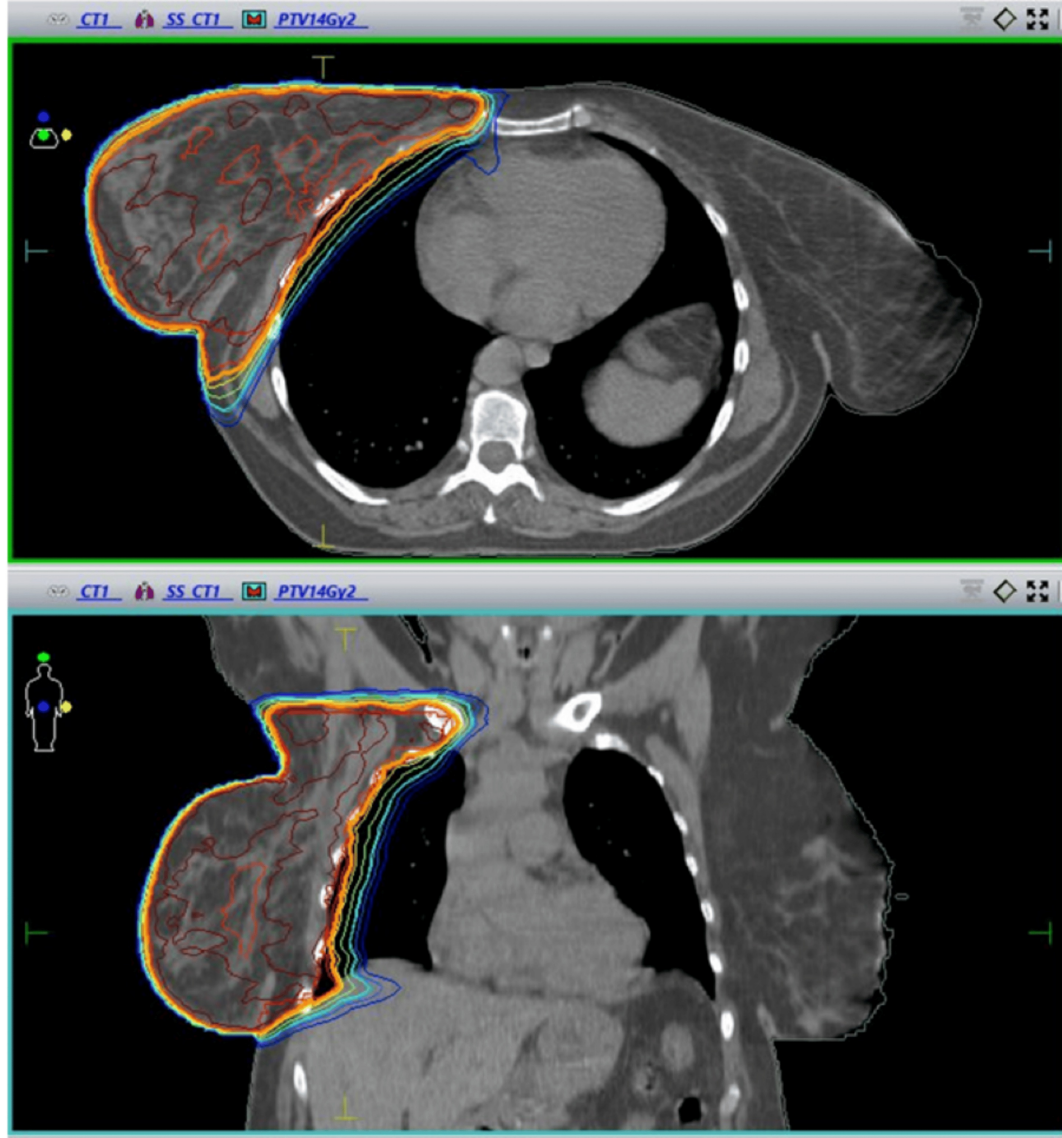
CT scan showing tumor formation.

We present a 50-year-old female patient referred to us by the Clinic of Radiation Oncology for mild radiodermatitis. The initial complaints were of a nodule in the area of the right breast in the upper outer quadrant, which the patient palpated. A full body CT scan with contrast was performed with data for a small tumor formation (Figure 1). The patient was staged as T1N0M0 and, after biopsy, underwent a sectoral surgical excision of a tumor of the right breast (lumpectomy) and axillary lymph node dissection with 11 negative lymph nodes. The tumor was histologically proved as invasive ductal carcinoma with size 0.6 cm, IHC: E-cadherin (+ve), ER (+ve) in 80%, PR (+ve) in 20%, Her 2 (-ve) 1+, Ki-67. Three weeks after that, the patient was started on postoperative radiotherapy to the area of the remaining breast and chest wall. RT was performed on linear accelerator Elekta Infinity with a daily dose of 2 Gy to a total prescribed dose of 50 Gy. The breast was immobilized by an individual thermoplastic mask and was treated with volumetric modulated arc therapy. It is important to note that the volume of the breast is 2988 cm^3,^ which is a prerequisite for more side effects and radiodermatitis.

 Dose distribution in all critical organs is presented in Table 1.

**Table 1:** Radiotherapy dose distribution. PTV: planning target volume.

Structure	Volume (cm³)	Min. dose (cGy)	Max. dose (cGy)	Mean dose (cGy)	Cold ref. (cGy)	Volume	Volume	Hot ref. (Gy)	Volume >(cm³)	Volume >(%)	% in Volume	Is in SS	Heterogeneity index	Conformity index
PTV	2877.798	40.592	56.657	50.814	47.5	61.801	2.15	53.5	133.265	4.63	100	yes	1.11	0.66
Lung right	2150.274	2.568	54.102	14.99	-	-	-	20	587.489	27.32	100	yes	13.12	0.02
Liver	2187.096	0.124	39.593	6.142	-	-	-	-	-	-	100	yes	60.94	0
Heart	1027.947	3.591	32.34	9.24	-	-		25	4.92	9.48	100	yes	4.45	0.01
Oesophagus	31.122	3.26	19.988	7.192	-	-	-	15.111	4.668	15	100	yes	5.17	-
Lung left	1725.036	1.41	17.866	5.032	-	-	-	17.358	0.035	0	100	yes	3.72	-
Breast sin	2924.988	0.735	9.825	2.488	-	-	-	9.585	0.035	0	100	yes	4.24	-
Spinal cord	65.442	1.421	12.239	3.49	-	-	-	11.885	0.035	0.5	100	yes	4.3	-
Tune liver	1766.85	0.124	21.021	4.127	-	-	-	-	-	-	100	yes	50.2	-
Skin (Unsp.Tiss.)	25684.077	0	55.375	4.531	-	-	-	-	-	-	-	-	184.79	0.88
Trachea	33.81	3.845	23.798	13.264	-	-	-	19.225	5.072	15	100	yes	3.46	0.35

**Figure 2:**
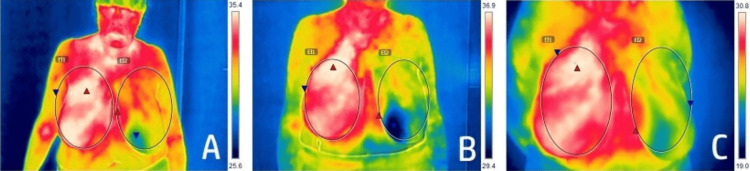
Changes in the skin temperature evaluated by thermography (A, B, C).

On the first visit to the Clinic of Dermatology and Venereology, the patient presented with mild radiodermatitis in the area of the right breast following ten fractions of RT. We performed a thermography, which showed a mild elevation of the temperature of the affected area. Five days after the visit, the patient was referred to us again with complaints of burning pain and a vesicular eruption in the previously affected area. We did a second thermography examination, which showed further elevation of the temperature (Figure 2).

**Figure 3:**
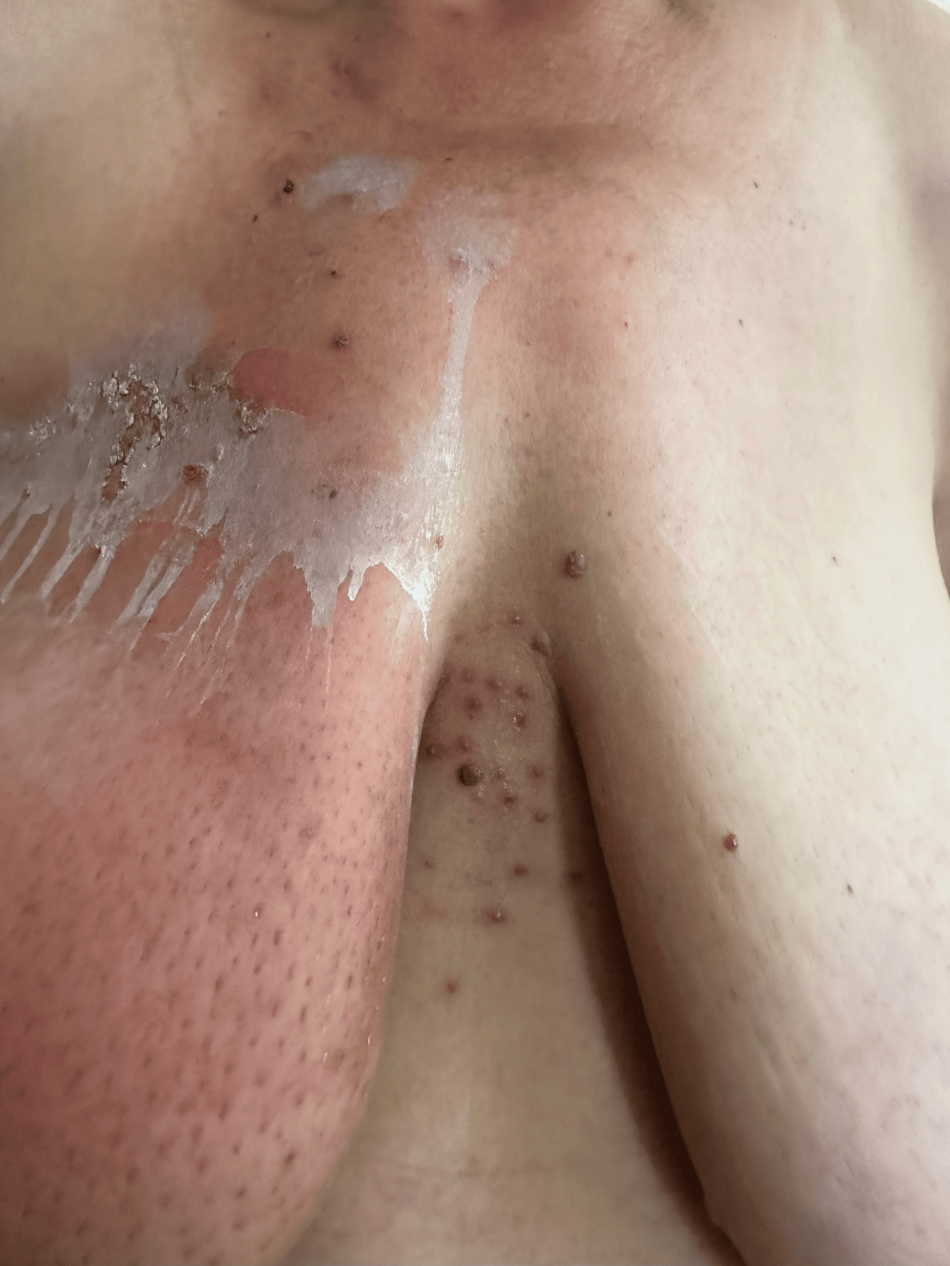
Herpetic rash in the area of the right breast.

A clinical diagnosis of Herpes zoster was made, and therapy with 5x400 mg Acyclovir daily was administered, and a recommendation was to temporarily discontinue RT (Figure 3). Ten days after the start of the treatment, the patient presented with a visibly improved dermatological status; no new vesicles, single exudating erosions and crusts, erythema was still visible. The patient continued therapy with a maintenance dose of Acyclovir to avoid reactivation of HZ and to continue RT without further interruption. 

## Discussion

Increasing age is the leading risk factor for HZ and is attributed to the immunosenescence of cell-mediated immunity, specifically to the VZV. Complications such as postherpetic neuralgia are also more common in elderly patients. Other risk factors include age, sex, family history of zoster, race, immunocompromised conditions, and multiple chronic conditions [7,8]. Patients suffering from at least one of the following conditions: asthma, chronic heart disease, chronic obstructive pulmonary disorder (COPD), depression, and rheumatoid arthritis, have a 30% increased risk of suffering from acute HZ [9]. 

The risk of developing HZ infection is known to be higher in patients with malignancies. In general, elderly cancer patients run a 1.2-2.4 times higher risk of developing HZ than those without cancer. Patients with hematological malignancies are at a higher risk of HZ development compared to patients with solid tumors [5,6]. The risk between HZ and RT has not been studied in detail. Dunst J et al. (2000) followed up on 1155 breast cancer patients who received postoperative radiotherapy. In 41 of those patients, HZ was observed, mostly in the first 2 years after the completion of radiotherapy. They reported that there was no correlation with other prognostic factors such as age, menopausal status, stage of disease, or the use of adjuvant chemotherapy. There was no link between the degree of acute skin reaction in the treated area and the occurrence of HZ [10].

Qian J et al. (2019) conducted a retrospective study and concluded that the HZ risk among participants with solid cancer receiving chemotherapy was greater than that of those without a chemotherapy record. Individuals who had not undergone CT or RT had the same risk of HZ as the general population. Among the patients receiving CT, RT was not associated with HZ development. Therefore, they concluded that the increased risk might be assigned to CT [4]. Lee PY et al. (2021) did a survey to determine the risk and time trends of HZ among patients with head and neck cancer, with or without RT. A total of 2160 patients were enrolled in the study. HZ was more commonly observed in cancer patients than in non-cancer patients, but with no significant difference. Patients undergoing RT were at more risk of suffering from HZ than the non-radiotherapy cohort. The 5-year incidence rates in the radiotherapy and non-radiotherapy cohorts were 8.9% and 5%, respectively [11].

Shimizuguchi T et al. (2020) conducted a retrospective cohort study to determine the role and impact of RT on the development of HZ. They found that the RT group had a significantly higher risk of developing HZ than the non-RT group. It is important to note that in 74 of the 120 patients who developed HZ after RT, HZ lesions appeared within the RT field [12]. The appearance of the herpetic lesions in the radiation field gives us reason to think that in our patient, the RT treatment is the main reason for the occurrence of HZ. Furthermore, most studies indicate a lower risk of HZ in cancer patients who do not undergo RT compared to those who do.

Some studies also show the significance of the radiation field on the risk of developing HZ during RT. Patients receiving limited-field RT had half the risk of HZ compared to those with extended-field RT. This indicates that more aggressive RT treatment is associated with a higher risk [13]. Cancer patients suffering from HZ are also at a higher risk of complications. Among HZ cases, 19% with hematologic malignancies and 14% with solid tumors suffered from postherpetic neuralgia for at least 30 days. Non pain-related complications in those groups were 30% and 18%, respectively.

The risk for postherpetic neuralgia is significantly higher in patients with HZ after RT. The duration of the pain is also longer in such patients and can last for more than 4 months in 18% of the patients, despite appropriate therapy. Some authors even recommend vaccination against zoster in patients aged 60 years or older. In the case described by us, the patient did not have severe pain but complained of burning and tightening of the skin in the affected area. In some patients, the typical severe pain is absent, especially when antiviral and anti-inflammatory therapy is started early. This highlights the importance of early diagnosis in such patients. Cancer patients suffering from HZ are also at a higher risk of complications. Among HZ cases, 19% with hematologic malignancies and 14% with solid tumors suffered from postherpetic neuralgia for at least 30 days. Non pain-related complications in those groups were 30% and 18%, respectively [3].

The risk for postherpetic neuralgia is significantly higher in patients with HZ after RT. The duration of the pain is also longer in such patients and can last for more than 4 months in 18% of the patients, despite appropriate therapy. Some authors even recommend vaccination against zoster in patients aged 60 years or older [14,15]. In the case we described, the patient did not have severe pain but complained of burning and tightening of the skin in the affected area. In some patients, the typical severe pain is absent, especially when antiviral and anti-inflammatory therapy is started early. This highlights the importance of early diagnosis in such patients.

## Conclusions

With the advance of treatment options for malignancies, we see fewer side effects from RT. However, patients with cancer have a higher risk of developing different infectious diseases, such as fungal, bacterial, or viral, including HZ. In patients with comorbidities and those of older age, it is good to consider the possibility of the occurrence of HZ and other bacterial and fungal infections, which may interfere with the treatment of the primary disease. In cancer patients with RT, such side effects can cause interruptions and prolongations of the radiotherapeutic course. In the early stages, HZ may be mistaken for acute or late radiodermatitis, leading to wrong treatment. It is important to raise awareness of the higher risk and proper management of HZ during RT in cancer patients in order to provide the best treatment care for patients in such a high-risk group.
